# Coordinated Transport by Dual Humanoid Robots Using Distributed Model Predictive Control

**DOI:** 10.3390/biomimetics9060332

**Published:** 2024-05-30

**Authors:** Shengjun Wen, Zhaoyuan Shi, Hongjun Li

**Affiliations:** 1 School of Zhongyuan-Petersburg Aviation, Zhongyuan University of Technology, Zhengzhou 450007, China; li.hongjun@zut.edu.cn; 2 School of Electronic Information, Zhongyuan University of Technology, Zhengzhou 450007, China

**Keywords:** dual-robot system, distributed model predictive control, trajectory tracking, collaborative transportation

## Abstract

Dual humanoid robot collaborative control systems possess better flexibility and adaptability in complex environments due to their similar structures to humans. This paper adopts a distributed model predictive controller based on the leader–follower approach to address the collaborative transportation control issue of dual humanoid robots. In the dual-robot collaborative control system, network latency issues may arise due to unstable network conditions, affecting the consistency of dual-robot collaboration. To solve this issue, a communication protocol was constructed through socket communication for dual-robot collaborative consistency, thereby resolving the problem of consistency in dual humanoid robot collaboration. Additionally, due to the complex structure of humanoid robots, there are deficiencies in position tracking accuracy during movement. To address the poor accuracy in position tracking, this paper proposes a distributed model predictive control that considers historical cumulative error, thus enhancing the position tracking accuracy of dual-robot collaborative control.

## 1. Introduction

Humanoid robots represent a complex dynamical system characterized by multiple variables, nonlinearity, strong coupling, and variable structures. They are an important branch in the field of robotics research. Compared to single-robot technology, multi-robot systems have numerous advantages. They operate within a broader workspace, offer greater maneuverability, and possess higher degrees of freedom, which can compensate for the limitations of individual robots and solve a wider range of tasks. In recent years, this area has become a significant focus within the realms of intelligent control and autonomous unmanned systems. With advancing research, the application scenarios and functions of multi-robot cooperative control continue to expand. Currently, multi-robot collaborative control is widely applied to complex tasks such as drone swarm control [1], formation control [2], and multi-robot target tracking [3]. They have become key technologies for collaborative transportation, surveillance search, and autonomous driving, holding substantial academic importance and practical value. The current multi-robot collaborative control algorithms encompass a variety of methods, including robust control [4], consensus control [5], linear quadratic regulator control [6], and reinforcement learning [7]. Comparatively, model predictive control (MPC) [8] can easily handle control constraints and perform rolling optimization. Through an internal model, MPC predicts the future performance of the system and solves for a suboptimal solution within a shorter time horizon, continuously updating it. Therefore, MPC can progressively adjust for model errors in real time, reducing the discrepancy between model outputs and references, ensuring the effective implementation of the control law. In multi-input multi-output (MIMO) systems like multi-robot collaboration, MPC can achieve better application.

Model predictive control (MPC) is an advanced control method that offers several advantages: (1) it is applicable to multi-input multi-output systems; (2) it exhibits strong robustness and stability; and (3) it can handle a variety of constraint conditions, among others. Currently, numerous researchers have employed MPC in cooperative control issues. SHEN et al. [9] designed a trajectory tracking control strategy based on the analysis of dynamic characteristics of autonomous underwater vehicles. By employing a distributed MPC approach, they decomposed a large optimization problem into several smaller subproblems and processed them separately, significantly reducing the number of floating-point operations required. Mohseni et al. [10] proposed a cooperative control strategy for the decentralized optimization control problem of autonomous vehicle systems, aimed at performing various complex traffic maneuvers. They then used a nonlinear MPC algorithm to solve it, incorporating collision avoidance constraints to ensure safety among vehicles.

Distributed model predictive control (DMPC), as a variant within MPC, has garnered widespread attention due to its flexible architecture, lower computational costs, and reduced communication requirements, among other advantages. Cao et al. [11] designed a robust DMPC considering external disturbances, introducing robust constraints to handle external perturbations, and analyzed the stability of the strategy as well as the overall multi-agent system. Wei et al. [12] proposed a robust DMPC method for a class of heterogeneous autonomous ground vehicles with input constraints and bounded external disturbances. By solving the DMPC problem, the optimal nominal control inputs were obtained. Dai et al. [13], considering the challenges of disturbances and coupling constraints to distributed control algorithms, proposed a distributed robust MPC algorithm for multi-agent systems with external disturbances and obstacle avoidance constraints. Pan et al. [14] proposed a DMPC scheme for maintaining the desired formation while a virtual leader tracks the reference trajectory. A virtual lead was designed to follow the reference path, with other agents maintaining the required distances and angles relative to the virtual lead to achieve trajectory tracking, overcoming the disadvantage where the failure of the traditional leader in a leader–follower model could destabilize the entire system.

The problem of robotic collaborative transport is one of the main application scenarios for robot coordination control. It plays a significant role across various industrial sectors. Robotic cooperative transport strategies are broadly divided into two categories: leader–follower [15] and encirclement cooperation. The leader–follower approach is primarily used in the coordination between two robots, where the leader plans the movement path and the follower maintains a specific relative positional relationship with the leader through certain constraint conditions. Munir et al. [16] addressed the consensus problem in leader–follower multi-robot cooperative systems by proposing an innovative arbitrary order finite-time sliding mode control design, which ensured the enforcement of sliding mode in finite time and maintained the stability of error dynamics. The effectiveness of the proposed approach was corroborated through rigorous closed-loop stability analysis and simulation examples. Ullah et al. [17] adopted a leader–follower strategy and proposed an innovative design for a distributed fixed-time synchronization controller based on neuro-adaptive non-singular terminal sliding mode control for higher-order multi-agent nonlinear systems. The effectiveness of this approach was demonstrated through simulation examples. Zhang et al. [18] adopted the leader–follower strategy, transforming the formation control problem into a follower’s trajectory tracking issue with respect to a virtual leader. They achieved position control among robots and verified the system’s stability using a Lyapunov function, ultimately enabling the transport of large items. Liu et al. [19], in the realm of control strategies, put forth a collaborative model that embodies the concept of a virtual leader and actual follower, segmenting the overall system’s control architecture into distinct hierarchies for the leader and follower robots. Additionally, they implemented a dual closed-loop kinematic control framework, transmuting the motion management of both robotic categories into trajectory tracking control problems. The efficacy of the proposed structure and control mechanism was corroborated through empirical testing. Wu et al. [20] investigated the guidance control method for multi-robot collaborative transportation of large components. They proposed various transportation configurations, including monocular–multidrive, binocular–bidrive, and binocular–multimotion–pair omnidirectional systems, along with motion control models. Additionally, they designed a path-tracking control method based on a leader–follower strategy, which incorporates homogeneous and heterogeneous architectures involving fuzzy logic and model predictive control. A prototype system was developed to conduct experiments, confirming the efficacy of the proposed methods.

In the problem of collaborative control of robots, most scholars have used wheeled robots as the research object, while less research has been carried out on the collaborative handling control of humanoid robots. Therefore, this paper establishes a dual humanoid robot collaborative handling control system. Due to the communication delay caused by network fluctuations in the process of dual-robot communication, which affects the consistency of dual-robot collaboration, this paper constructs a consensus communication protocol to solve the consistency problem of robot collaboration. Further investigation has revealed that model predictive control (MPC) methods, as utilized in the referenced literature, commonly exhibit deficiencies in control precision and lack the requisite level of accuracy. To address the issue of insufficient control precision within the dual-humanoid robot collaborative control system, this paper proposes a DMPC approach that takes into account historical cumulative error. This method is devised to enhance the steady-state performance in the collaborative control of the dual robots, thereby significantly improving control precision and enhancing accuracy.

## 2. Robot Motion Model

### 2.1. Humanoid Robot Kinematics Analysis

**Figure 1:**
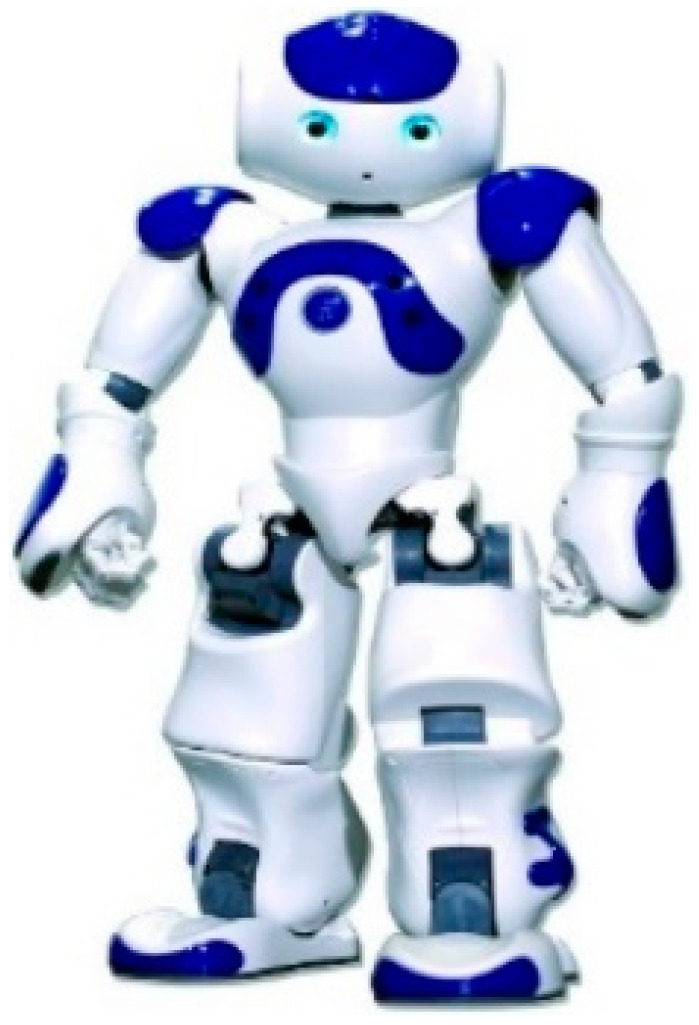
NAO robot.

This paper employs the NAO robot as the research platform, as illustrated in Figure 1. The NAO robot [21], produced by Aldebaran Robotics, is a bipedal intelligent robot measuring 58 cm in height and weighing 5.4 kg. It features a total of 25 degrees of freedom, and its hardware components include a CPU, ultrasonic sensors, gyroscopes, and infrared devices, among others, as detailed in Table 1, which presents an overview of some of the NAO robot’s hardware specifications.

The NAO robot features a hand with three fingers capable of performing various grasping and transporting tasks. The motions of the robot are actuated by brushless DC motors. The motor specifications for the finger joints include a no-load speed of 8400 RPM (revolutions per minute) and a rated torque of 4.9 mNm (millinewton meters), while the motor specifications for the arm joints include a no-load speed of 10,700 RPM and a rated torque of 6.2 mNm.

**Figure 2:**
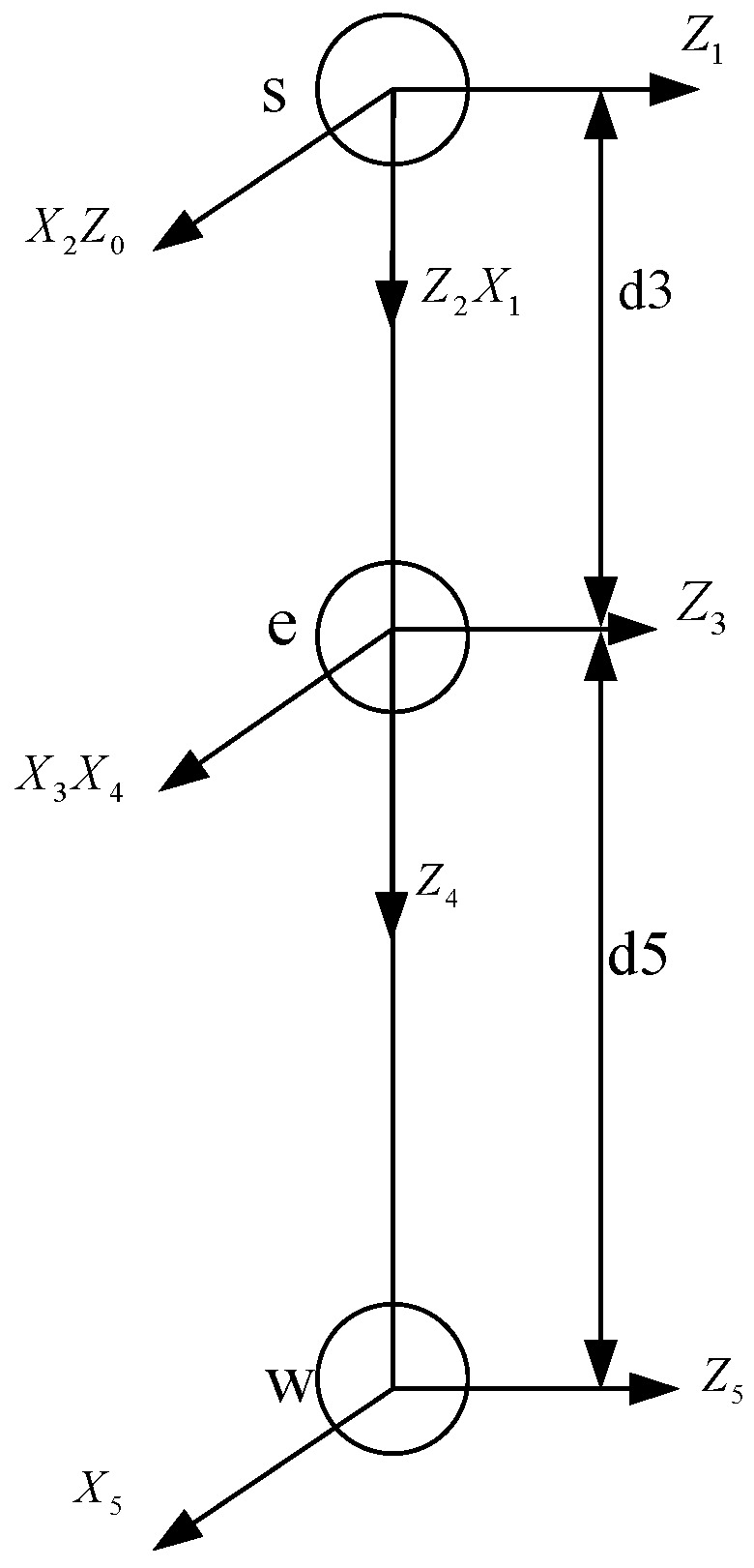
NAO robot right arm D-H model.

The right arm of the NAO robot, for instance, possesses five degrees of freedom and is a type of serial kinematic structure. According to the Denavit–Hartenberg (D-H) convention [22], the data presented in Table 2 can be derived. On this basis, a linkage coordinate system for the NAO robot’s right arm is established. As shown in Figure 2, s, e, and w represent the shoulder joint, elbow joint, and wrist joint of the NAO robot, respectively. The dimensions of the links are obtained through the parameters of the NAO robot’s right arm, where d3 = 90 mm and d5 = 108.55 mm.

**Figure 3:**
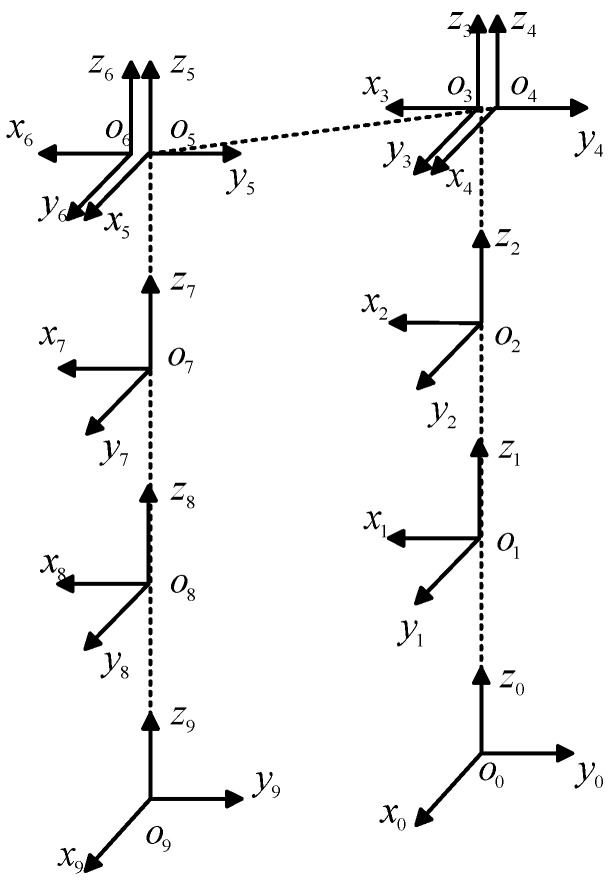
NAO robot leg D-H model.

The NAO robot has five degrees of freedom in each leg, with four force sensors installed on the sole of each foot. The forward, lateral, and vertical directions of the robot are defined as the X-axis, Y-axis, and Z-axis directions, respectively. When the robot is standing, the origin of the world coordinate system is located at the center position between the robot’s two feet. To facilitate subsequent analysis, a D-H model is established for the robot’s legs, as shown in Figure 3.

When constructing the model for the supporting leg, taking the left leg as an example, let O_0_ be the base coordinate system and O_4_ be the end-effector coordinate system. Using this as a reference, the position matrix is obtained by incorporating the translational and rotational transformation matrices along the X-axis and Z-axis into the homogeneous transformation matrix defined by the D-H method, as shown in Equation (1).

(1) $$A_{i}=\left[\begin{array}{cccc} \cos\theta_{i} & -\sin\theta_{i}\sin\alpha_{i} & \sin\theta_{i}\sin\alpha_{i} & a_{i}\cos\theta_{i}\\ \sin\theta_{i} & \cos\theta_{i}\cos\alpha_{i} & -\cos\theta_{i}\sin\alpha_{i} & a_{i}\sin\theta_{i}\\ 0 & \sin\alpha_{i} & \cos\alpha_{i} & d_{i}\\ 0 & 0 & 0 & 1 \end{array}\right]$$

In Equation (1), $a_{i}$ represents the link length, $\alpha_{i}$ represents the link twist angle, $d_{i}$ represents the distance between joints, and $\theta_{i}$ represents the joint twist angle. Based on the chain rule of homogeneous transformation, the homogeneous transformation matrix of *O*_4_ relative to *O*_0_ is derived, as shown in Equation (2). R3×340 is the rotation matrix, and P3×140 is the position vector.

(2) T40=A1A2A3A4=R3×340P3×14003×311×3

When constructing the swing leg model, using the left leg as an example, let *O*_0_ be the end-effector coordinate system and *O*_4_ be the base coordinate system. It is easy to derive the homogeneous transformation matrix. Due to the symmetry between the left and right legs, when the right leg is the swing leg, the homogeneous transformation matrix of *O*_9_ relative to *O*_5_ can be determined as matrix T59, which is similar in form to matrix T40. Similarly, when the right leg is used as the supporting leg, the homogeneous transformation matrix of *O*_5_ relative to *O*_9_ can be determined as matrix T59.

### 2.2. NAO Robot Kinematics and Dynamics Modeling

**Figure 4:**
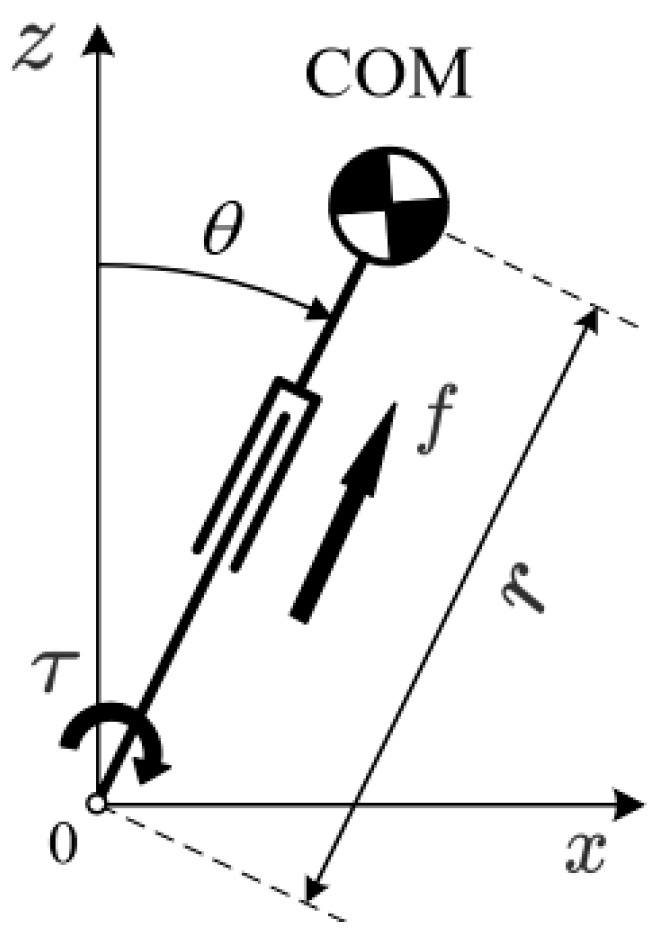
Linear inverted pendulum model.

The robot’s walking is simplified to a linear inverted pendulum model for dynamic analysis [23], as shown in Figure 4. In this model, the robot’s trunk is simplified to a point mass COM that concentrates all the mass, and the robot’s leg is simplified to a massless, telescopic link of length *r* connecting the trunk and the foot. τ represents the torque at the robot’s ankle, and *f* represents the reactive force received by the robot when pushing off the ground. Subsequently, the motion of the center of mass is driven through the bending and stretching of the legs.

After the initial state is determined, the motion state of the inverted pendulum can be described as shown in Equation (3), where $T_{c}$ is a constant ratio of gravitational acceleration to the height of the center of mass; $x_{0}$ and ${\dot{x}}_{0}$ are, respectively, the displacement and velocity of the center of mass in the X direction at zero time, that is, the initial conditions.

(3) $$\left\{\begin{array}{c} x_{t}=x_{0}\cosh(t/T_{c})+T_{c}{\dot{x}}_{0}\sinh(t/T_{c})\\ {\dot{x}}_{t}=x_{0}/T_{c}\sinh(t/T_{c})+{\dot{x}}_{0}\cosh(t/T_{c})\\ T_{c}=\sqrt{z/g} \end{array}\right.$$

**Figure 5:**
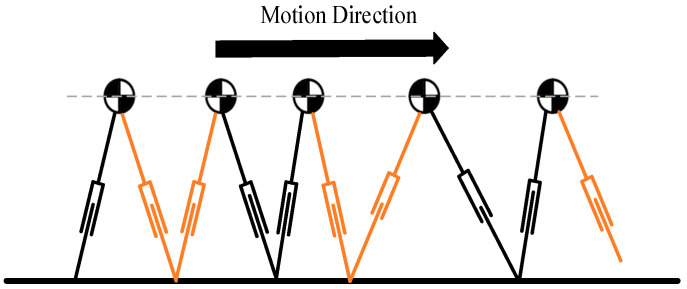
Linear inverted pendulum gait diagram.

The humanoid robot can be simplified to an inverted pendulum model, with the robot’s movement illustrated in Figure 5. During locomotion, the robot’s center of mass moves horizontally, and ground contact is managed by alternating leg support, ensuring that there is always one leg in contact with the ground.

### 2.3. State Space Equation Based on ZMP

For bipedal robots, maintaining balanced and stable walking is very important. The ZMP (zero moment point) [24] is the criterion for static or dynamic stable walking of bipedal robots. Its essence is to calculate the combined force point of the ground on the robot under the condition that the robot will not topple over, and when the robot has acceleration, the ground’s acting force balances with gravity and inertial force at the ZMP. Since gravity remains constant, when the height of the center of mass does not change, the position of the ZMP corresponds to the motion acceleration of the center of mass. The support point of the linear inverted pendulum is its ZMP. Therefore, the mathematical expression of ZMP can be analyzed as shown in Equation (4).

(4) $$\left\{\begin{array}{c} \ddot{x}=\frac{g}{z_{c}}x-\frac{1}{mz_{c}}\tau_{x}\\ \ddot{y}=\frac{g}{z_{c}}y-\frac{1}{mz_{c}}\tau_{y} \end{array}\right.$$

In the equation, *m* represents the mass of the center of gravity, *g* represents the constant of gravitational acceleration, and $\tau_{x}$ and $\tau_{y}$, respectively, represent the torques of the inverted pendulum model rotating around the X-axis and Y-axis. The position equation of ZMP for a robot under the constraints of horizontal plane motion is shown in Equation (5).

(5) $$\left\{\begin{array}{c} p_{x}=-\frac{\tau_{y}}{mg}\\ p_{y}=\frac{\tau_{x}}{mg} \end{array}\right.$$

Taking the derivative of acceleration ${\dddot{u}}_{x}$ as the input to the ZMP equation, and considering horizontal displacement x, velocity $\dot{x}$, and acceleration $\ddot{x}$ as state variables, combined with Equations (1)–(3), we can obtain the state space representation of the ZMP equation, as shown in Equations (6) and (7).

(6) $$\frac{d}{dx}\left[\begin{array}{c} x\\ \dot{x}\\ \ddot{x} \end{array}\right]=\left[\begin{array}{ccc} 0 & 1 & 0\\ 0 & 0 & 1\\ 0 & 0 & 0 \end{array}\right]\left[\begin{array}{c} x\\ \dot{x}\\ \ddot{x} \end{array}\right]+\left[\begin{array}{c} 0\\ 0\\ 1 \end{array}\right]u_{x}$$

(7) $$p_{x}=\left[\begin{array}{ccc} 1 & 0 & -z_{c}/g \end{array}\right]\left[\begin{array}{c} x\\ \dot{x}\\ \ddot{x} \end{array}\right]$$

After obtaining the state space equations in the continuous time domain, when using model predictive control methods, it is necessary to perform discretization. Setting the sampling time as $T_{s}$, the velocity of the center of mass in the horizontal direction at the $k^{th}$ sampling instance is shown in Equation (8). The acceleration in the horizontal direction is shown in Equation (9).

(8) $$\dot{x}(k)=\frac{x(k+1)-x(k)}{T_{s}}$$

(9) $$\ddot{x}(k)=\frac{\dot{x}(k+1)-\dot{x}(k)}{T_{s}}$$

In these equations, x(k) is the horizontal position of the centroid at the $k^{th}$ sampling instant; x(k+1) is the horizontal position of the centroid at the ${\left(k+1\right)}^{st}$ sampling instant. $\dot{x}(k)$ and $\dot{x}(k+1)$ represent the velocity of the centroid’s horizontal movement at the $k^{th}$ and ${\left(k+1\right)}^{st}$ sampling instances, respectively; $\ddot{x}(k)$ is the acceleration in the horizontal direction of the centroid at the $k^{th}$ sampling instance.

Let the state variables be defined as displacement, velocity and acceleration, represented as $X(k)={\left[x(k),\dot{x}(k),\ddot{x}(k)\right]}^{T}$; with the second derivative of velocity as the control input $u(k)=\dddot{x}(k)$, and the output as the ZMP trajectory $p(k)=x_{zmp}(k)$ of the NAO robot, the state-space equation of the system can be represented by Equation (10).

(10) $$\left\{\begin{array}{c} X(k+1)=AX(k)+BU(k)\\ P(k)=CX(k) \end{array}\right.$$

In Equation (10):

$$A=\left[\begin{array}{ccc} 1 & T_{s} & T_{s}^{2}/2\\ 0 & 1 & T_{s}\\ 0 & 0 & 1 \end{array}\right];\;B={\left[\begin{array}{ccc} T_{s}^{3}/6 & T_{s}^{2}/2 & T_{s} \end{array}\right]}^{T};\;C=\left[\begin{array}{ccc} 1 & 0 & -z_{c}/g \end{array}\right].$$

## 3. Dual Robot Cooperative Control

### 3.1. Dual-Robot Communication System

The research objective of this paper is to design a DMPC controller to address the cooperative control issue of bipedal humanoid robot systems, thereby achieving consensus on trajectory tracking and position control. Before establishing the cooperative control system model for the dual robots, it is first necessary to establish a communication system between the robots.

**Figure 6:**
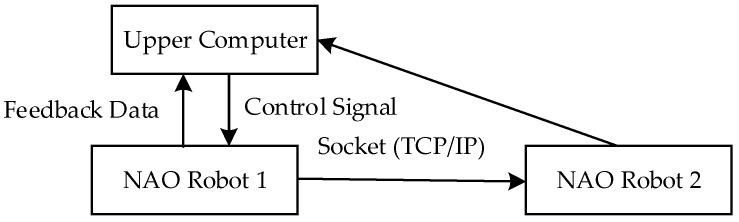
Dual-robot system communication diagram.

This research employs the TCP/IP [25] communication protocol to achieve inter-robot communication. To establish the communication diagram shown in Figure 6, where robot1 acts as the leader robot and robot2 as the follower robot, robot1 receives control signals from the higher-level controller and sends commands to robot2, which receives and transmits information back to robot1.

Therefore, we can obtain the adjacency matrix of the dual-robot system, as shown in Equation (11).

(11) $$A=\left[\begin{array}{ccc} 0 & 0 & 0\\ 1 & 1 & 0\\ 0 & 1 & 0 \end{array}\right]$$

In the context of dual-robot communication, to address network communication delays, which may mitigate the impact of communication delays on coordination consistency, a consensual communication protocol is established based on the collaborative actions of the dual-robot system, as shown in Table 3. Initially, action commands for the robots are pre-planned and set according to task requirements, including actions such as object grasping and coordinated movement. Subsequently, the actions to be executed by the robots, along with their respective start execution times, are compiled into structured data, with multiple structures forming an array that represents a series of consistent actions. Following confirmation of the accuracy of the structured array, the leader robot transmits a sequence of action commands to the follower. Ultimately, upon reaching the designated time points, both the leader and follower initiate the corresponding action commands to execute the planned actions. This approach ensures the consistency of collaboration between the two robots.

### 3.2. Leader DMPC Controller Design

MPC predicts system behavior and performs optimization through a predictive model, thus obtaining an optimal control sequence at each time step [26]. Therefore, the construction of the prediction model is very important for the entire control allocation process, and its accuracy and applicability largely determine the overall performance of the system.

The obtained discrete state-space equation is shown in Equation (10). Given $p^{ref}(k)$ as the reference position for the ZMP, in order to make the system’s output p(k) track the target ZMP position $p^{ref}(k)$ as accurately as possible, we define a cost function to serve as the loss function, thereby constructing an optimization problem. The cost function is shown in Equation (12).

(12) $$J=\sum_{i=k}^{\infty }\left[Q_{e}e{\left(i\right)}^{2}+\Delta X^{T}(i)Q_{x}\Delta X(i)+R\Delta u^{2}(i)\right]$$

where $e(i)\equiv p(k)-p^{ref}(k)$ represents the trajectory error, ΔX(k)≡X(k)−X(k−1) is the increment of the state vector, and Δu(k)≡u(k)−u(k−1) is the increment of the input. The weights $Q_{e}$, R and $Q_{x}$ stand for the ZMP trajectory error, minimum control effort, and state variables, respectively, and $Q_{e}>0$ and R>0. At any time k, the solution u(k) that minimizes J is the optimal solution required.

At each sampling time, if the reference values of the ZMP for the next $N_{L}$ steps can be known in advance, then the optimal controller that minimizes the evaluation index is shown in Equation (13).

(13) $$u(k)=-G_{i}\sum_{i=0}^{k}e(k)-G_{x}X(k)-\sum_{j=1}^{N_{L}}G_{p}(j)P^{ref}(k+j)$$

In the equations, $G_{i}$, $G_{x}$, and $G_{p}$ represent the corresponding gains for the error, state variables, and output. As shown in Equations (14)–(16), the parameters can be solved using the dare() function in MATLAB, and the model predictive control is realized through continuous iteration.

(14) $$G_{i}=A^{T}G_{i}A-A^{T}G_{i}B{\left(B^{T}G_{i}B+R\right)}^{-1}B^{T}G_{i}A+Q_{x}$$

(15) $$G_{x}=eig(A-B\times G_{p})$$

(16) $$G_{p}={\left(B^{T}G_{i}+R\right)}^{-1}B^{T}G_{i}A$$

### 3.3. Follower DMPC Controller Design

**Figure 7:**
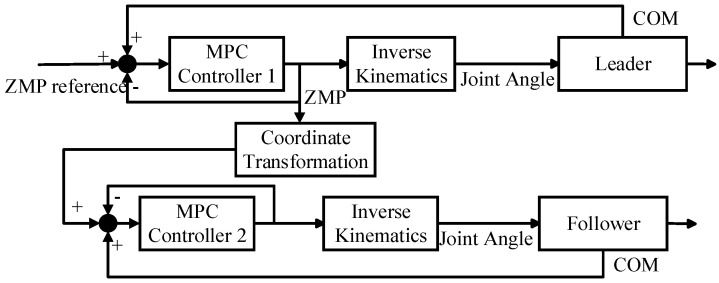
Leader−follower robots cooperative control block diagram.

To establish the distributed control block diagram based on MPC control as shown in Figure 7, first, a ZMP reference trajectory is input to the leader. After going through the MPC controller, an optimal control sequence for the $k^{th}$ moment is obtained. Then, by solving the robot’s inverse kinematics, motion control of the leader is achieved. The ZMP values output by the leader are then transformed into secondary coordinates and used as the follower’s reference trajectory. Subsequently, through the MPC controller, the follower tracks the leader’s trajectory, thus realizing the coordinated position control of the dual robots.

Since the two robots stand face-to-face while performing tasks, their paths are not exactly the same, and the follower’s reference path is the actual trajectory of the leader robot. Therefore, it is necessary to perform a secondary coordinate transformation on the leader’s movement trajectory to use it as the reference path for the follower robot.

Relative to the follower robot’s own coordinate system, the reference trajectory is shown in Equation (17). Here, $P_{1}^{ref}$ represents the reference trajectory for the follower, $P_{1}$ represents the actual movement trajectory of the leader robot, and $R_{z}$ denotes the rotation matrix around the Z-axis.

(17) $$P_{1}^{ref}=-P_{1}R_{z}=-P_{1}\left[\begin{array}{ccc} \cos\theta & \sin\theta & 0\\ -\sin\theta & \cos\theta & 0\\ 0 & 0 & 1 \end{array}\right]$$

In the dual-robot collaborative task, the control objective of the follower robot is to autonomously control its movement path to maintain relative direction and position with the leader. In the system, the state space equations for the follower and leader robots are the same and can be represented by Equation (18):

(18) $$\left\{\begin{array}{c} X_{1}(k+1)=AX_{1}(k)+BU_{1}(k)\\ P_{1}(k)=CX_{1}(k) \end{array}\right.$$

The dual-robot formation matrix is shown in Equation (19).

(19) $$D=\left[\begin{array}{cc} 0 & \Delta x\\ 0 & \Delta y\\ 0 & \Delta v\\ 0 & \Delta a \end{array}\right]$$

The first column of the matrix represents the motion state information of the leader robot relative to itself, with all elements being zero. The second column of the matrix represents the motion state of the follower with respect to the leader robot in the system, containing four rows of data: relative longitudinal coordinate difference, relative lateral coordinate difference, relative velocity error, and relative acceleration error.

The weighted sum of the formation error output between the follower and the leader robots at time k is shown in Equation (20).

(20) $$error(k)=\sum_{j}^{3}a_{3j}[P(k)-P_{1}(k)+\Delta ]$$

In Equation (20), $a_{3j}$ represents the specific elements of the adjacency matrix A, which reflects the communication relationships between robots in the dual-robot system, as established by Equation (11); Δ represents the position, velocity, and acceleration errors between the leader robot and the follower robot, which can be obtained by subtracting the second column from the first column in the formation matrix D.

When designing the DMPC controller for coordinated motion, the follower must maintain consistent displacement, velocity, and acceleration with the leader. Thus, the cost function for the follower at time k can be designed as shown in Equation (21).

(21) $$J_{1}=\sum_{i=k}^{\infty }\left[Q_{e}error{\left(i\right)}^{2}+\Delta {X_{1}}^{T}(i)Q_{x}\Delta X_{1}(i)+R\Delta {u_{1}}^{2}(i)\right]$$

In Equation (21), $Q_{e}$, R and $Q_{x}$ represent the state error weight, minimum control weight, and state error weight, respectively. The first and second terms on the right side of the equation represent the error between the follower and the leader in the coordinated control and the state error output of the follower, respectively, while the third term is the weighted sum of changes in control inputs.

By constructing the follower’s cost function, the problem can be transformed into a Riccati equation to solve. Similarly, the MATLAB function dare() can be used to calculate the weights. Thus, the mathematical model of the follower’s DMPC (dynamic movement profile criterion) controller is obtained as shown in Equation (22).

(22) $$u_{1}(k)=-G_{i}\sum_{i=0}^{k}error_{1}(k)-G_{x}X_{1}(k)-\sum_{j=1}^{N_{L}}G_{p}(j){P_{1}}^{ref}(k+j)$$

## 4. Experimental Results and Analysis

### 4.1. Collaborative Control Experimental Design

The dual-humanoid robot system exhibits higher flexibility and adaptability in complex environments. This paper is based on the premise that dual-humanoid robots can conduct rescue operations for victims in complex situations such as natural disasters and accident scenes with casualties. An experiment was designed for the dual-humanoid robots to cooperatively carry a stretcher, aiming to validate the feasibility of the DMPC controller.

**Figure 8:**
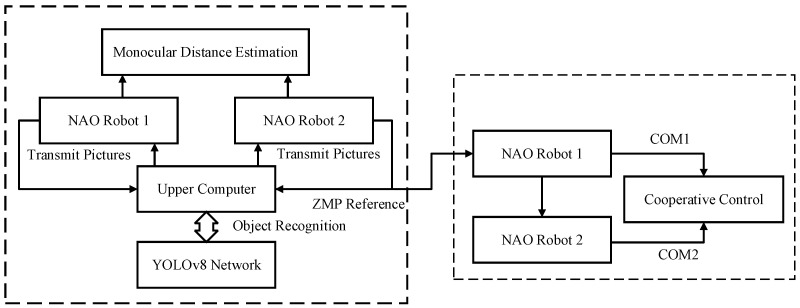
Overall experimental design.

Figure 8 illustrates the overall experimental design framework. Initially, the two robots identify the stretcher using their onboard visual sensors. Subsequently, they measure the distance between themselves and the stretcher using a monocular ranging algorithm and move close to the stretcher. During locomotion, errors may occur due to factors such as slippery ground surfaces and motor aging. Therefore, upon reaching the designated spot, the robots adjust their postures. After posture adjustment, the follower robot sends feedback to the leader, employing the NAO robot’s built-in setAngles() function to control the arm movements and simultaneously perform the grasping task. Finally, the two robots lift the stretcher together, with the follower tracking the leader’s trajectory to reach the target location.

### 4.2. Object Positioning and Grasping

This experiment involves collecting 300 images of the target stretcher from different angles using the NAO robot’s lower camera and processing them with rotations, mirroring, etc. Then, the Yolov8 network is trained for a total of 800 rounds.

**Figure 9:**
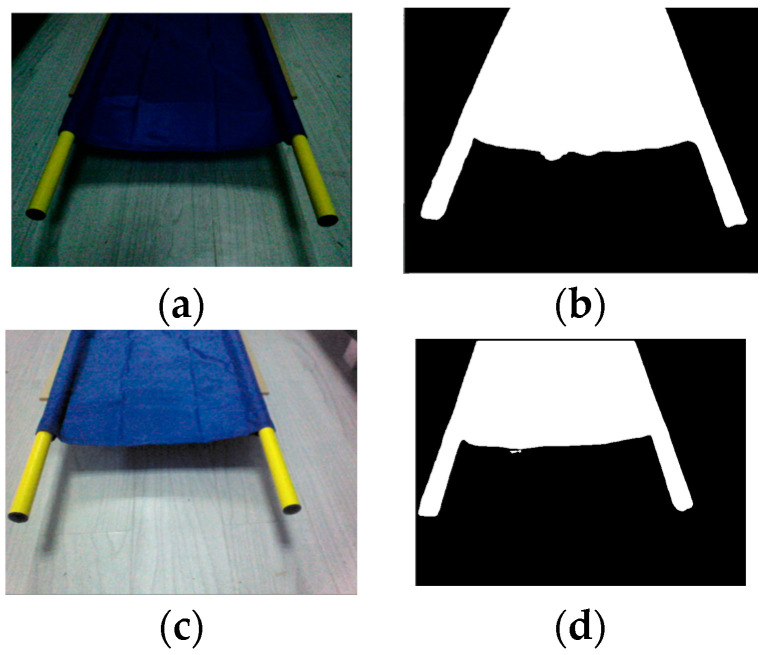
Target recognition: (**a**) original image captured by leader; (**b**) leader target recognition results; (**c**) original image captured by follower; (**d**) follower target recognition results.

Firstly, using the lower cameras of the NAO robots, the two robots capture raw images as shown in Figure 9a,c and send them back to the control computer. The Yolov8 network in the Python3 environment is then used to identify the target pole pieces. The binary images of the target objects are transmitted to Python2 via Socket communication using the TCP/IP protocol, as shown in Figure 9b,d.

Based on the results shown in Figure 9b,d, monocular ranging is performed using the center point of the line connecting the two handles at each side of the stretcher as the target point. After obtaining the distances in the X and Y directions relative to the robot itself, it moves close to the target stretcher.

Upon the robots reaching the designated positions and after postural adjustments, the handle positions are measured again, and the arms are controlled to perform the grasping action. During the grasping process, the end-effectors of the arms employ a motion strategy of acceleration-constant speed-deceleration through the setAngles() function, ensuring that the NAO robot’s hands can smoothly reach the target position.

**Figure 10:**
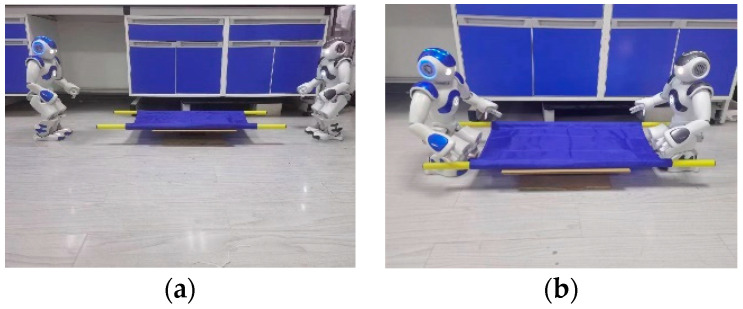
Object positioning and grasping process: (**a**) two robots positioning towards the target stretcher; (**b**) two robots grasping the target stretcher.

In the robot target positioning and grasping experiment, as shown in Figure 10a,b, the blue NAO robot acts as the leader, while the grey NAO robot is the follower. Both robots use monocular vision to identify and locate the stretcher simultaneously and move to the designated positions. After adjusting their postures, they determine the grasping locations and control the end-effectors of their arms to reach the grasping points.

### 4.3. Dual-Robot Collaborative Transportation Experiment

**Figure 11:**
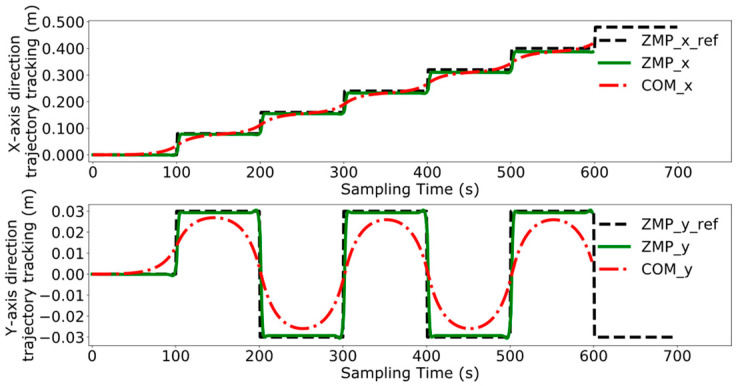
Time-domain ZMP trajectory tracking under current error.

**Figure 12:**
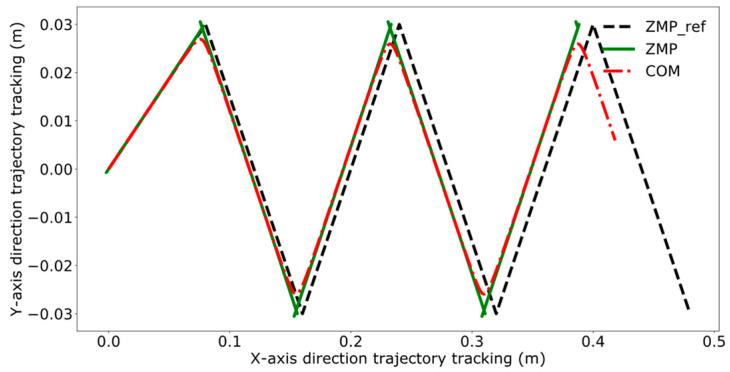
ZMP trajectory tracking under current error.

**Figure 13:**
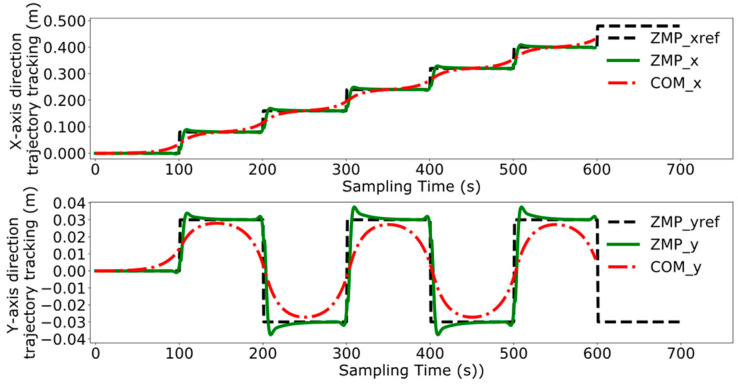
Time-domain ZMP trajectory tracking under accumulated error.

**Figure 14:**
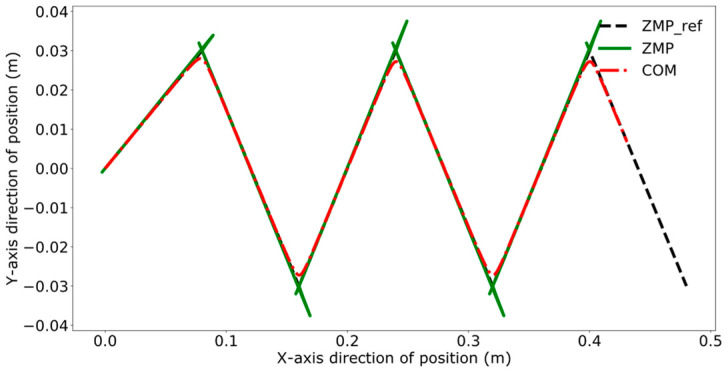
ZMP trajectory tracking under accumulated error.

Reference [24] proposes. a model predictive control method for bipedal robots based on a five-centroid model. However, reference [24] does not consider the error effect caused by historical cumulative error in MPC control, which leads to the lack of trajectory tracking control accuracy. Therefore, this paper proposes a DMPC control method based on error accumulation to compare and analyze with the method adopted in reference [24]. A coordinate system for the dual-robot system is established with the leader’s position as the origin. In the xoy plane, the leader’s coordinates are (0,0), and the follower’s initial position is (1 m, 0). The reference trajectory for ZMP is set as [(0,0), (0.08,0.03), (0.16,−0.03), (0.24,0.03), (0.32,−0.03), (0.40,0.03), (0.48,−0.03)]. At point (0,0), the robot maintains a bipedal standing position, followed by alternating left and right leg movements, with the coordinates in the reference trajectory representing the landing points of the left and right feet, respectively. The leader’s ZMP trajectory tracking was compared using the control method employed in reference [24] and the error accumulation-based DMPC control method proposed in this paper, with the experimental results presented in Figure 11, Figure 12, Figure 13 and Figure 14, which include the ZMP trajectory of the robot in the X and Y directions as well as the trajectory of the robot’s center of mass during locomotion.

Figure 11 and Figure 12 illustrate the results of tracking the ZMP trajectory using the MPC control method proposed in reference [24]. Figure 11 shows the movement of the robot’s center of mass in the X and Y directions in the time domain. The red line represents the trajectory of the center of mass, the black line indicates the reference trajectory, and the green line denotes the leader’s tracking trajectory. Figure 12 shows the result of the robot’s trajectory tracking on the XY plane for the robot in the leader’s coordinate system.

Figure 13 and Figure 14 demonstrate the experimental results obtained by using the error accumulation-based DMPC control method proposed in this paper. Figure 13 displays the tracking of the trajectory in the X and Y directions in the time domain, considering the cumulative error. Figure 14 presents the outcome of the robot’s trajectory tracking under the leader’s coordinate system based on accumulated error.

Comparing Figure 11 and Figure 13, the method used in reference [24] fails to perfectly track the ZMP reference trajectory in both the X and Y axis directions during the steady-state phase. In contrast, the method proposed in this paper achieves better tracking of the reference trajectory once the system reaches the steady state, enhancing the steady-state performance of the system.

**Figure 15:**
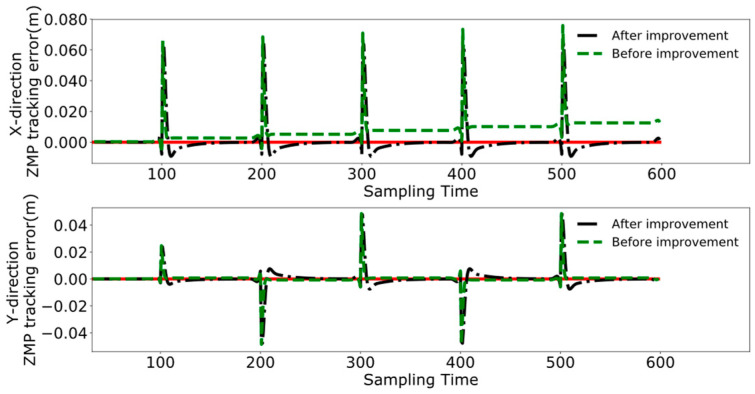
Leader’s trajectory tracking error.

A comparative analysis between Figure 12 and Figure 14 reveals that the method utilized in reference [24] exhibits increasing tracking error with the increment of displacement. In contrast, the results depicted in Figure 14 indicate that the DMPC control method, which considers cumulative errors, consistently maintains effective tracking of the reference trajectory. Figure 15 more vividly illustrates the error variation between the two methods. The method used in reference [24] fails to accurately track the ZMP trajectory in the X-axis direction, whereas the improved DMPC control method proposed in this paper can accurately and stably track the trajectory, demonstrating superior control precision. The abrupt changes observed in the figure are due to the leg exchange during the robot’s walking process.

To achieve position coordination control between two robots, the follower robot employs the trajectory of the leader robot transformed through Equation (10) as its reference trajectory. Since the robots are facing each other during the collaborative task, in the follower’s coordinate system, the follower is moving in the negative direction of the X-axis.

**Figure 16:**
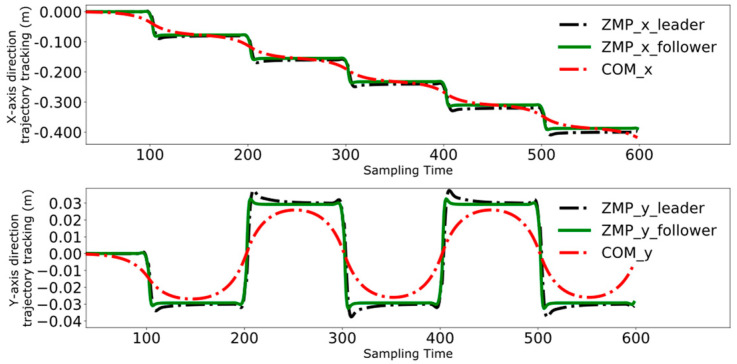
Pre-improvement ZMP trajectory tracking of the follower in the time domain.

**Figure 17:**
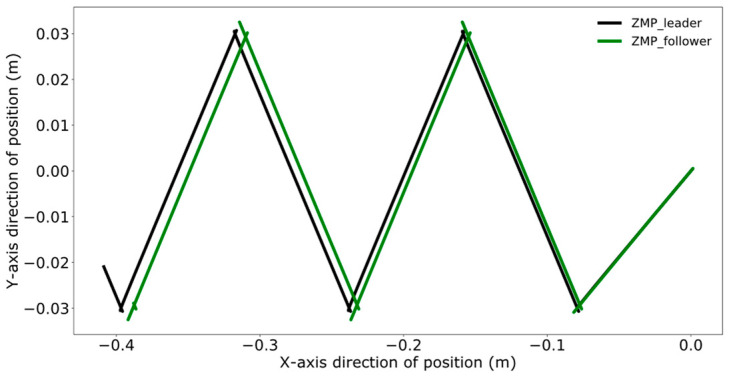
Pre-improvement follower’s ZMP trajectory tracking.

**Figure 18:**
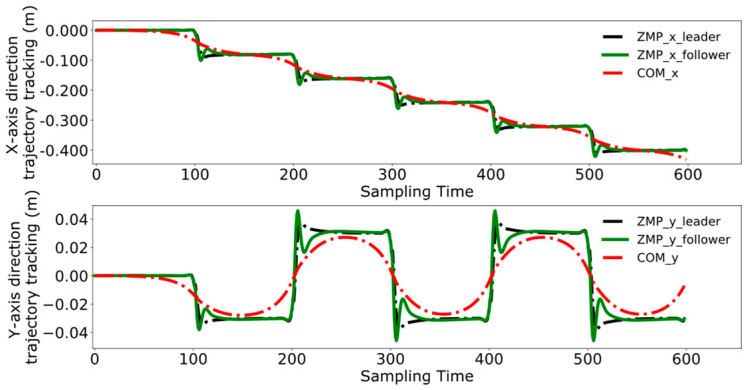
Post-improvement ZMP trajectory tracking of the follower in the time domain.

**Figure 19:**
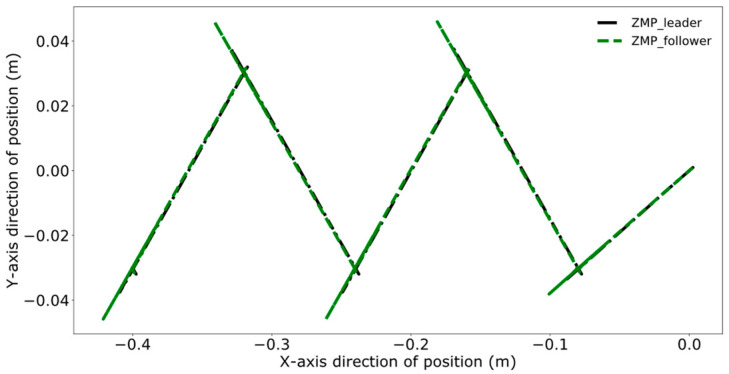
Post-improvement follower’s ZMP trajectory tracking.

Figure 16, Figure 17, Figure 18 and Figure 19 present the tracking performance of a follower robot using the leader’s ZMP trajectory as a reference, comparing two different methods. Figure 16 and Figure 17 utilize the method from reference [24], while Figure 18 and Figure 19 display the results from the improved DMPC controller. Comparing the outcomes for both control methods on the follower reveals a pattern similar to that observed with the leader. The improved DMPC controller shows significantly improved tracking accuracy, whereas the unmodified method continues to exhibit an increase in tracking error with displacement, ultimately reaching an error margin of approximately 2 cm. This further corroborates the efficacy of the improved DMPC controller in accurately following the leader’s ZMP trajectory.

The two methods are integrated into the leader–follower collaborative control framework designed in this paper, with the forward direction of the leader robot designated as the positive X-axis and from right to left as the positive Y-axis. Since the two robots face each other during transportation, when the leader robot steps forward with its left leg, the follower robot steps forward with its right leg, ensuring alternating progress that maintains consistency of the center of mass along the Y-axis, thereby enhancing stability during transport. Given that the stretcher being carried is 1 m in length, it is necessary for the two robots to maintain a distance of approximately 1 m in the X direction throughout the collaborative carrying process.

**Figure 20:**
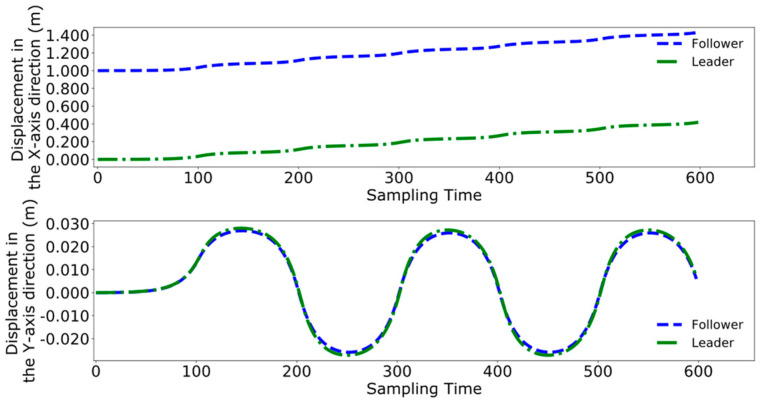
Pre-improvement dual-robot centroid displacement.

**Figure 21:**
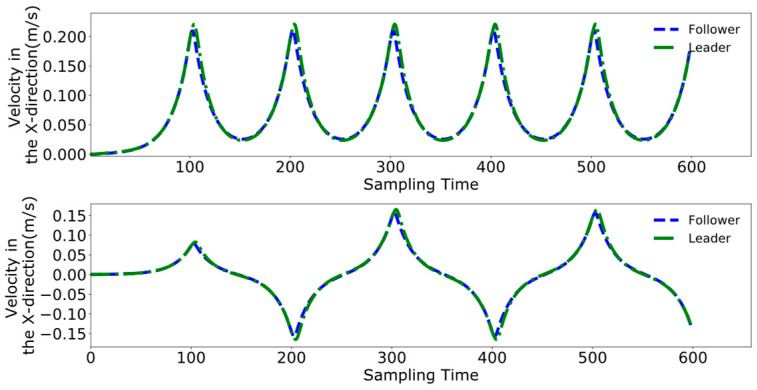
Pre-improvement dual-robot centroid velocity.

**Figure 22:**
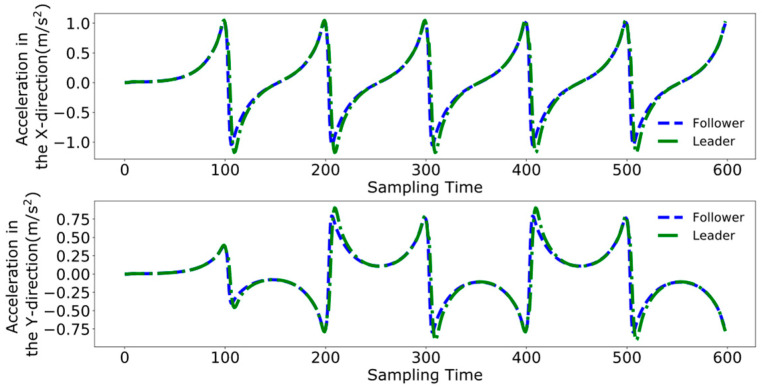
Pre-improvement dual-robot centroid acceleration.

Initially employing the method used in reference [24], Figure 20, Figure 21 and Figure 22, respectively, represent the collaborative control of the displacement, velocity, and acceleration of the center of mass for the leader and follower. The blue lines indicate the results of the follower’s center of mass movements, while the green lines denote the movements of the leader.

**Figure 23:**
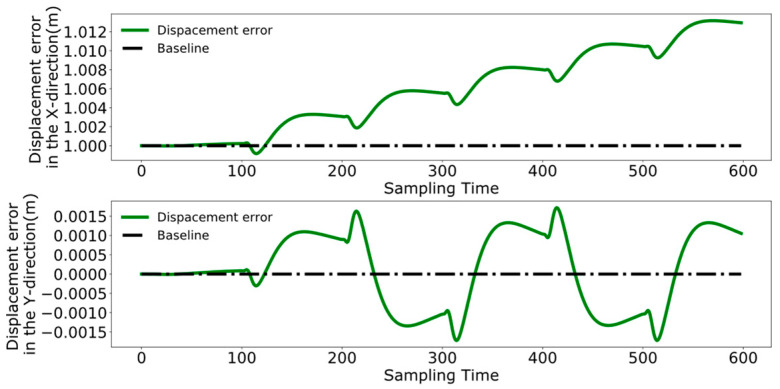
Pre-improvement dual-robot centroid displacement error.

**Figure 24:**
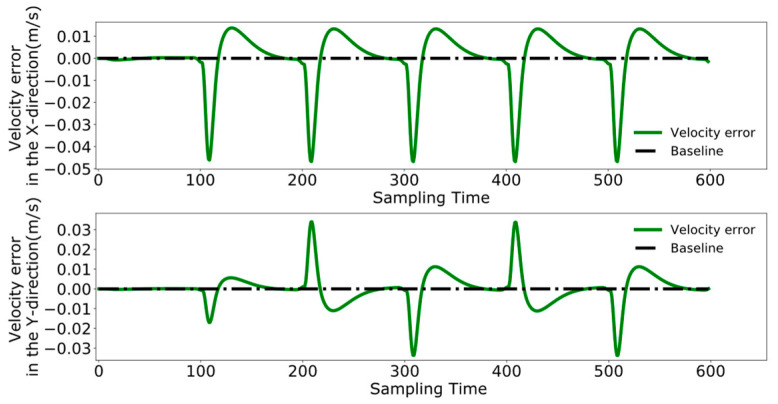
Pre-improvement dual-robot centroid velocity error.

**Figure 25:**
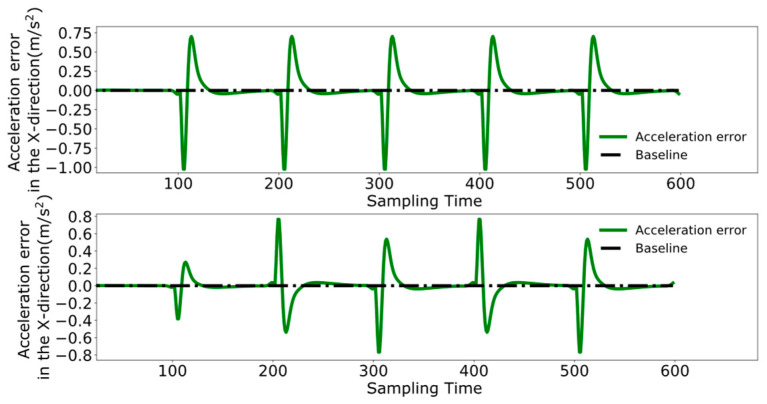
Pre-improvement dual-robot centroid acceleration error.

To better analyze the coordination between the two robots, Figure 23, Figure 24 and Figure 25 display the displacement, velocity, and acceleration errors during the collaborative control process. Figure 23 illustrates the displacement errors in the X and Y directions for both robots, with the error in the X direction gradually increasing over time. The error in the Y direction oscillates around zero. Figure 24 shows the maximum errors in the centroid velocities of the two robots in the X and Y directions, with a maximum error of 0.05 m/s in the X direction and 0.03 m/s in the Y direction. Figure 25 presents the acceleration errors for both robots, with the maximum errors in the X and Y directions being 1 m/s^2^ and 0.7 m/s^2^, respectively.

**Figure 26:**
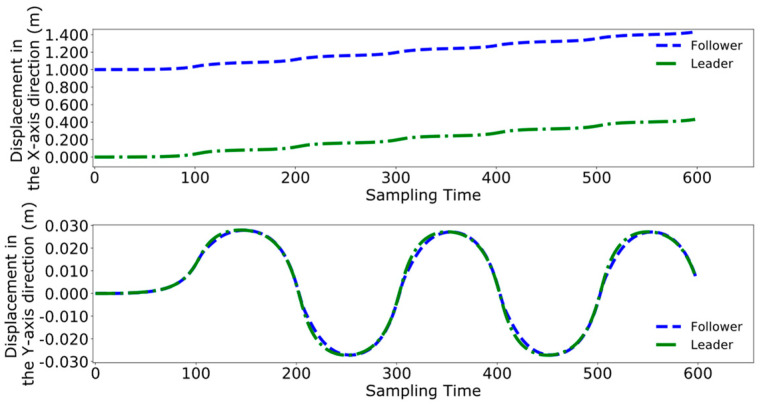
Post-improvement dual-robot centroid displacement.

**Figure 27:**
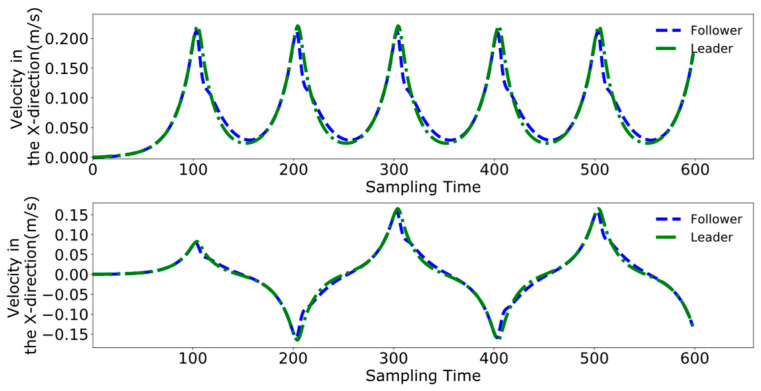
Post-improvement dual-robot centroid velocity.

**Figure 28:**
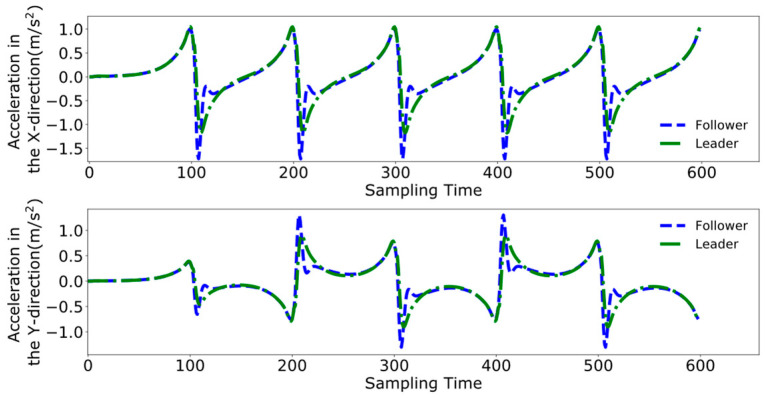
Post-improvement dual-robot centroid acceleration.

Figure 26, Figure 27 and Figure 28 demonstrate the collaborative control motions of the leader and follower based on the enhanced DMPC control method. Figure 26 illustrates the displacements in the X and Y directions for both robots, indicating that a consistent distance of 1 m in the X direction is maintained between them. Figure 27 and Figure 28, respectively, show the uniformity of the centroid velocity and acceleration in the X and Y directions for both robots during system control.

**Figure 29:**
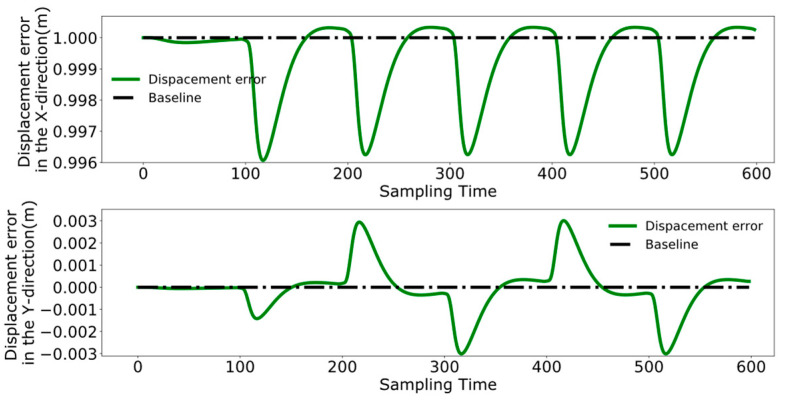
Post-improvement dual-robot centroid displacement error.

**Figure 30:**
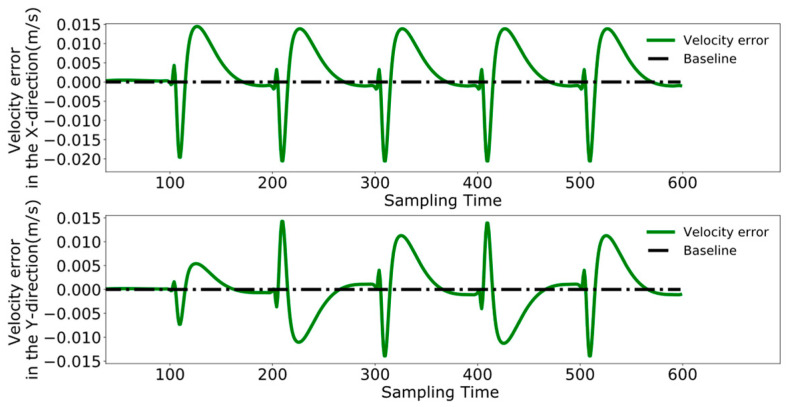
Post-improvement dual-robot centroid velocity error.

**Figure 31:**
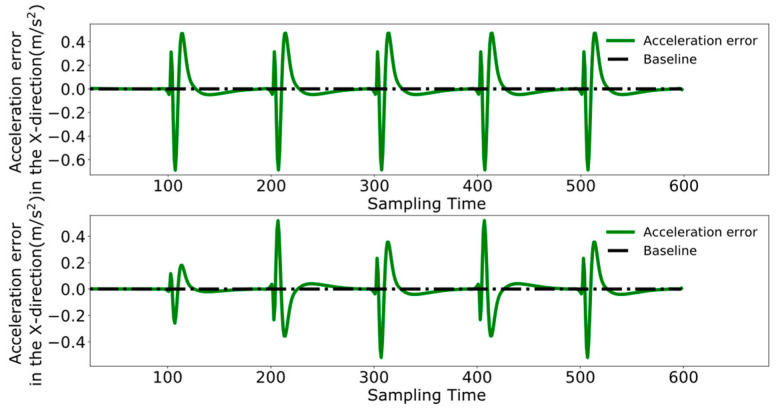
Post-improvement dual-robot centroid acceleration error.

Figure 29, Figure 30 and Figure 31, respectively, present the centroid displacement, velocity, and acceleration errors of the two robots based on the improved DMPC collaborative control proposed in this paper. Figure 29 displays the centroid displacement error, with a maximum error of 0.004 m in the X direction, which not only enhances control precision compared to the displacement error results shown in Figure 23 but also maintains the error around zero as time increases. Figure 30 shows the centroid velocity results of the two robots at the same moment, with a maximum error of 0.02 m/s in the X direction and 0.015 m/s in the Y direction. Compared to the maximum errors of 0.045 m/s and 0.03 m/s shown in Figure 24 for the X and Y directions, respectively, there is a significant improvement. Figure 31 illustrates the centroid acceleration collaboration results in the dual-robot system, with maximum errors of 0.6 m/s^2^ and 0.45 m/s^2^ in the X and Y directions, respectively. In comparison to Figure 25, there is an enhancement of 0.4 m/s^2^ in the X direction and 0.3 m/s^2^ in the Y direction.

Through a comparative analysis employing two methods, in this paper, certain improvements in control precision for both leader control and dual-robot collaborative control compared to the method presented in reference [24], enhancing the steady-state performance of the system, are shown. Additionally, by validating the errors in centroid displacement, velocity, and acceleration of the two robots using both methods, it is proven that the leader-follower collaborative control based on DMPC designed in this study is effective and feasible.

**Figure 32:**
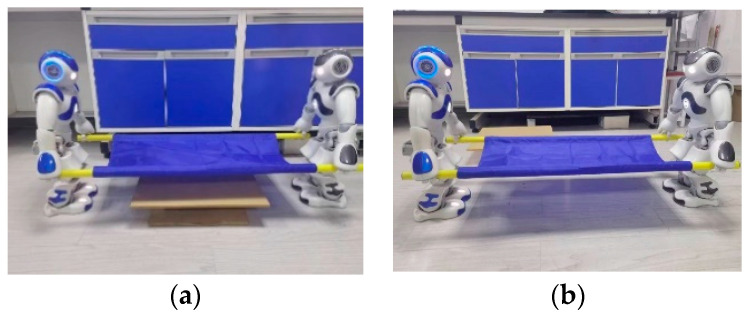
Collaborative transportation of dual humanoid robots: (**a**) two robots lifting the stretcher simultaneously; (**b**) robots cooperatively transporting a stretcher.

Figure 32 illustrates the dual-robot collaborative transport process, where they initially move simultaneously to the right side of the leader robot, circumventing the placement stage. Subsequently, they proceed in the positive X direction of the leader robot for transportation. During this process, the follower robot walks backward, continuously receiving positional information from the leader robot to ensure that the center of mass for both robots remains aligned in the X and Y directions within the leader robot’s coordinate system.

## 5. Conclusions

This paper presents a dual bipedal robot DMPC (Dynamic Motion Primitives Control) collaborative control system for carrying stretchers using two NAO robots against the backdrop of rescue robots at casualty accident sites. In the dual-robot collaborative control system, a dual bipedal robot consistency communication protocol is constructed to address the issue of consistency in collaborative control. Concurrently, to mitigate the significant trajectory tracking error of the dual-robot system, a DMPC control method considering cumulative error effects is proposed. Compared with existing methods, the proposed control approach demonstrates superior steady-state performance and enhanced control accuracy, with a tracking error within 0.01 m, whether in the individual control of leaders and followers or in the collaborative control of both robots. Furthermore, the dual-robot control system effectively coordinates the velocity and acceleration of the two robots, confirming the effectiveness of the established collaborative control system for dual bipedal robots.

Given that the environment at accident sites is generally complex, characterized by uneven terrain and numerous obstacles, this paper does not delve deeply into research on this aspect. Therefore, future work will focus on how to implement collaborative control of dual robots in complex environments. This could involve upgrading and adding sensor equipment to the robots, enabling them to better perceive their surroundings. Additionally, neural networks such as CNNs (convolutional neural networks) [27] and BP (backpropagation) can be utilized to enhance the intelligence of the robots, thereby strengthening their adaptability to various conditions.

## Figures and Tables

**Table 1:** NAO robot hardware parameters.

Hardware Components	Parameters
CPU	2 × Intel Atom Z530 Processors
Memory	1 GB RAM, 2 GB Flash Memory
Network Connection	Ethernet, Wi-Fi
Battery	Lithium Battery

**Table 2:** D-H parameters of the right arm of NAO robot.

Link	$\theta_{i}$/°	$d_{i}$/mm	$a_{i}$/mm	$\alpha_{i}$/°
1	$\theta_{1}$	0	0	90
2	$\theta_{2}$(−90°)	0	0	−90
3	$\theta_{3}$	d3	0	90
4	$\theta_{4}$	0	0	−90
5	$\theta_{5}$(−90°)	d5	0	90

**Table 3:** Consensus protocol.

Protocol Field	Data Type
Command Content	string
Execution Time	datetime

## Data Availability

Data are contained within the article.
